# Simulation and Theory of Antibody Binding to Crowded Antigen-Covered Surfaces

**DOI:** 10.1371/journal.pcbi.1004752

**Published:** 2016-03-11

**Authors:** Cristiano De Michele, Paolo De Los Rios, Giuseppe Foffi, Francesco Piazza

**Affiliations:** 1 Dipartimento di Fisica, “Sapienza” Università di Roma, Roma, Italy; 2 Institute of Theoretical Physics, Ecole Polytechnique Fédérale de Lausanne, Lausanne, Switzerland; 3 Laboratoire de Physique des Solides (LPS), UMR8502, Université Paris sud, Orsay, France; 4 Université d’Orléans, Centre de Biophysique Moléculaire, CNRS-UPR4301, Orléans, France; Bar Ilan University, ISRAEL

## Abstract

In this paper we introduce a fully flexible coarse-grained model of immunoglobulin G (IgG) antibodies parametrized directly on cryo-EM data and simulate the binding dynamics of many IgGs to antigens adsorbed on a surface at increasing densities. Moreover, we work out a theoretical model that allows to explain all the features observed in the simulations. Our combined computational and theoretical framework is in excellent agreement with surface-plasmon resonance data and allows us to establish a number of important results. (i) Internal flexibility is key to maximize bivalent binding, flexible IgGs being able to explore the surface with their second arm in search for an available hapten. This is made clear by the strongly reduced ability to bind with both arms displayed by artificial IgGs designed to rigidly keep a prescribed shape. (ii) The large size of IgGs is instrumental to keep neighboring molecules at a certain distance (surface repulsion), which essentially makes antigens within reach of the second Fab always unoccupied on average. (iii) One needs to account independently for the thermodynamic and geometric factors that regulate the binding equilibrium. The key geometrical parameters, besides excluded-volume repulsion, describe the screening of free haptens by neighboring bound antibodies. We prove that the thermodynamic parameters govern the low-antigen-concentration regime, while the surface screening and repulsion only affect the binding at high hapten densities. Importantly, we prove that screening effects are concealed in relative measures, such as the fraction of bivalently bound antibodies. Overall, our model provides a valuable, accurate theoretical paradigm beyond existing frameworks to interpret experimental profiles of antibodies binding to multi-valent surfaces of different sorts in many contexts.

## Author Summary

Antibodies are the main working horses of the human immune system. Remarkably, no matter the size or the shape of the pathological intruders, these extremely flexible three-lobe molecules are able to form a complex, thus eliciting an immune response. What makes antibodies so effective? To answer this and other questions, we have developed a simplified computational scheme to simulate the dynamics of many antibodies interacting with each other and with antigens. Coarse-grained models are a great opportunity, as they give access to a true multi-scale approach to biologically relevant problems. In this work, our innovative method allowed us to simulate the binding process of many antibodies to surface-adsorbed antigens. This led us to elucidate and quantify many important physical aspects of their biological function in agreement with experiments, such as the role of their flexibility and crowding effects at the hapten-covered surface, which were shown to finely regulate the avidity.

## Introduction

Because of their prominent role in the human immune system, antibodies are among the most important biomolecules. Like other large complex proteins, they are increasingly being exploited in modern nanobiotechnology [1] and biomedical [2] applications. Antibodies are large molecules, whose flexibility is deeply related to their function, granting them enhanced potency [3–6] and astonishing abilities, from binding an extremely diverse palette of antigens [7] to *walking* on antigen-covered surfaces [8]. In general, understanding the details of antibody flexibility and the associated limitations can inform the design of antiviral vaccines and therapies [3]. Unfortunately, simulating many large molecules interacting with one another is a challenging task at present, because even single, medium-size proteins can be simulated at atomistic resolution only for time scales that are several orders of magnitude shorter than the processes they are involved in [9]. Any description of more articulated systems, composed by several different proteins in mutual interaction goes beyond the possibilities of any detailed simulations. As a consequence, novel approaches are necessary that allow spanning longer timescales and accounting for more complex settings.

Coarse-graining (CG) has come to the fore in recent times as a promising strategy for the simulation of large proteins and of protein complexes [10–19]. A coarse-grained model is built by neglecting all details below a selected length scale. Residue based CG [20, 21], for example, describes amino-acids as simple beads of a radius that reproduces that of the original residues and positioned at the coordinates of the C_*α*_ atoms or of the amino-acid center of mass. Because of the massive reduction of degrees of freedom and the simplification of the corresponding force-fields, CG schemes can access much longer timescales, at the obvious price of a loss of detail. Yet, this is not necessarily a limitation, as long as such approaches aim at addressing phenomena whose length scale is consistent with the CG simplification of the system. Extreme applications of CG have, for example, made possible the simulation of a crowded cellular cytoplasm with the aim of estimating the diffusion constant of proteins [22, 23].

**Fig 1:**
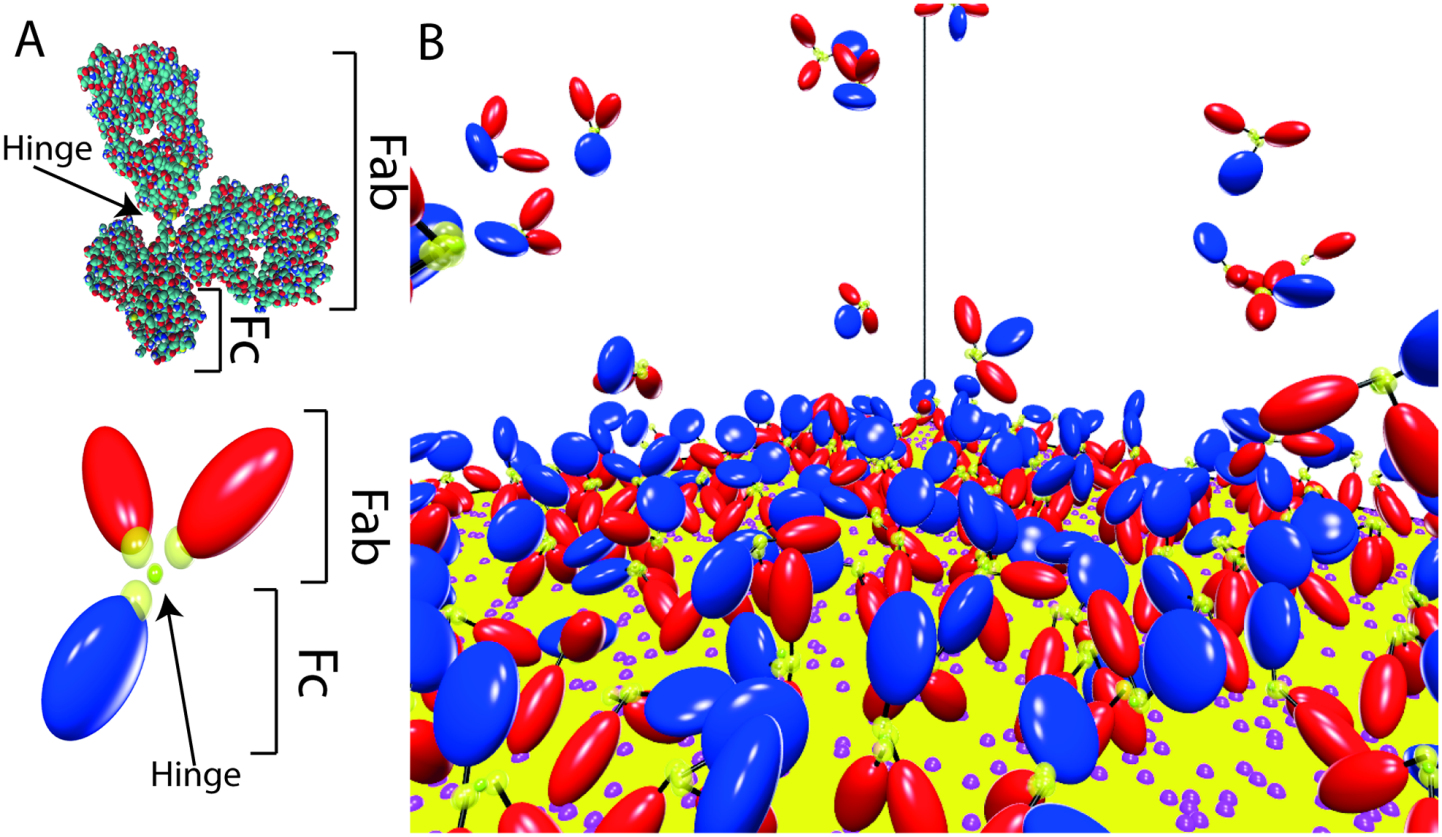
The coarse-grained model of IgGs. A) The two Fab domains are in red, while the Fc stem is shown in blue. The yellow transparent spheres picture attractive spherical domains (radial piece-wise potentials) that link together the three domains (in the hinge region) and allow binding an antigen (Fab tips). The T-shaped crystallographic structure 1IGY is also shown for comparison. B) Snapshot from our simulations, showing a close-up of the antigen-covered surface. Antigens are recognizable as pink spots on the surface.

In this work we introduce a novel CG model of IgG antibodies, which are large molecules composed of three domains [24–27]: two identical Fab arms, that bind antigens, connected to the Fc stem by a hinge region (Fig 1A). Our CG model is based on the results of recent cryo-electron tomography experiments [28, 29], and show that a careful reduction of the system complexity brings within reach a problem that would otherwise be intractable, namely the collective binding of antibodies to antigens distributed on a surface, with account of both the internal dynamics of IgGs and their mutual excluded volume on the surface. We use our results to validate an analytical model of the reaction kinetics that goes beyond the ones that have been proposed to date, and to provide a more rigorous interpretation of experiments from the literature.

## Results

### Coarse-grained modelling of IgG antibodies based on Cryo-ET experiments

A suitable modeling of IgGs has to take necessarily into account their great flexibility, which has been highlighted in several cryo-electron tomography (cryo-ET) [28, 29] and AFM [30] experiments.

**Fig 2:**
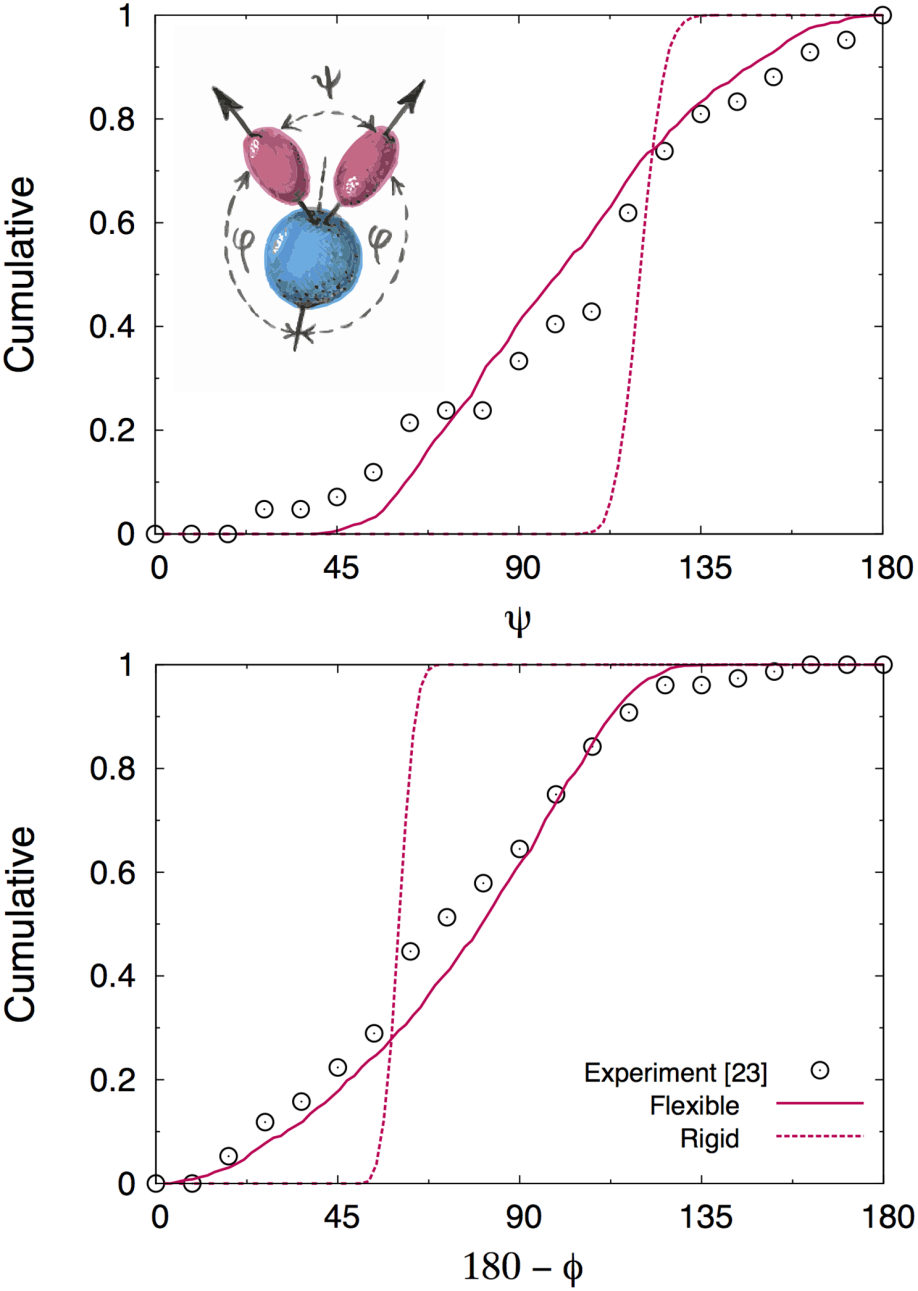
Cumulative distributions of the angles *ϕ* and *θ*. Symbols are the experimental cryo-ET results from [28]. Solid lines are the results of the EDBD simulations for our flexible and IgG models. Dashed lines refer to the rigid model, where the angles *ϕ* and *ψ* have been fixed at the value 2*π*/3. These curves show that our implementation of the fixed-angle constraint works fine (see methods).

In particular, Cryo-ET reconstructions have provided access to the probability distributions of the angle formed by the two Fab domains (*ψ*) and the one between Fab and Fc domains (*ϕ*, see Fig 2). Here we devise a coarse-grained model where each domain is described as a rigid hard body. More specifically, the three domains are modeled by revolution ellipsoids (Fig 1B), two prolate (Fabs) and one oblate (Fc), whose dimensions have been tuned so as to fit the hydrodynamic sizes measured in sedimentation experiments [31] (see Methods for more details). In close analogy with real antibodies, the three domains are joined by a flexible hinge designed so as to keep the three domains at distances compatible with the steric constraints that can be evinced from X-ray [27] and Cryo-ET experiments [28, 29]. The interaction site for antigen binding is described as a spherical surface (spot) on the tip of the Fab domains (see Fig 1B), which corresponds to a radial piece-wise constant attractive potential of finite range (see Methods for more details on the CG architecture, and Fig 1 for a comparison with the atomistic crystallographic structure, PDB 1IGY).

In our model, excluded volume is the only interaction between the three domains within a single IgG molecule. Nonetheless, Event-Driven Brownian dynamics (EDBD) simulations of a single antibody showed clearly that our flexible model is able to reproduce the experimental statistics of *ψ* and *ϕ* angles from Cryo-ET measurements (Fig 2). It is worth stressing that utterly reasonable angular distributions can be obtained solely by enforcing inter-domain steric effects that take into account (i) the correct *shape* of the three domains and (ii) the appropriate *size* of the hinge region. The weaker contributions from inter-domain potential energy terms highlighted in Ref. [28] thus only bring about minor modifications that can be safely ignored in the context of this work.

### Ensemble binding of IgG molecules to surface-adsorbed antigens

It has now been established experimentally that the binding of antibodies to antigens adsorbed on a surface is a complex phenomenon, with contributions from monovalent IgGs binding, *i.e*. by means of a single Fab arm, as well as from bivalent IgGs binding, where both Fab arms are bound, each to a different antigen [32]. The relative equilibrium weight of the two binding arrangements depends on the surface density of antigens and simplified kinetic models have been proposed in the literature [32, 33].

Our CG IgG model makes it possible to investigate *in silico* the behavior of ensembles of antibodies binding to a surface with surface-adsorbed antigens, reproducing exactly the experimental conditions found, *e.g*., in Ref [32]. Accordingly, we simulated *N*_0_ = 250 IgGs that freely diffuse in a box of side *L* and that can bind to antigens randomly distributed on the bottom surface at an assigned surface density *σ*. Assuming that the size of Fabs’ semi-axes is 1 nm, the concentration of IgG in our simulations box is 0.4 mM. Surface density of antigens typically ranges from 10^−9^ mol/m^2^ to 10^−7^ mol/m^2^, which are comparable with common values on cell surfaces (around 1.5 × 10^−9^ mol/m^2^[34]) and virus capsids (up to 10^−7^ mol/m^2^[35]). For influenza A virus, for example, the density is around 10^−8^ mol/m^2^, while for HIV is around 10^−10^ mol/m^2^[3], while it is between 10^−9^ and 10^−7^ mol/m^2^ on chips used in Surface plasmon resonance (SPR) experiments [32, 36].

The antigens interact with the Fab tips of incoming antibodies through square-well potentials of prescribed depth, which fixes the *k*_on_ and *k*_off_ rates of antigen-antibody binding kinetics. The molecular-level description of the system allows us to measure the precise number of monovalent (*N*_1_) and bivalent (*N*_2_) bound antibodies at equilibrium for each value of *σ*. Furthermore, we can gauge how the ability of antibodies to bind to the antigens is modulated by (i) intra-IgG flexibility and (ii) inter-IgG excluded volume. Further details are provided in the Methods section.

**Fig 3:**
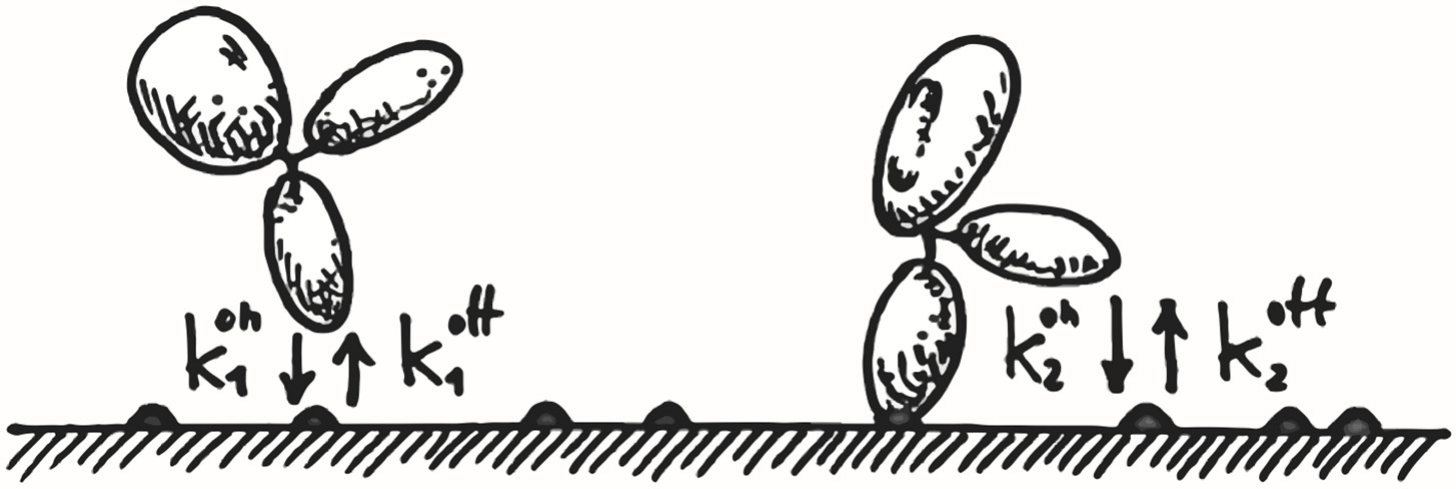
Cartoon illustrating the double-step kinetics of antibodies to surface-adsorbed antigens (dark spots on the surface).

**Fig 4:**
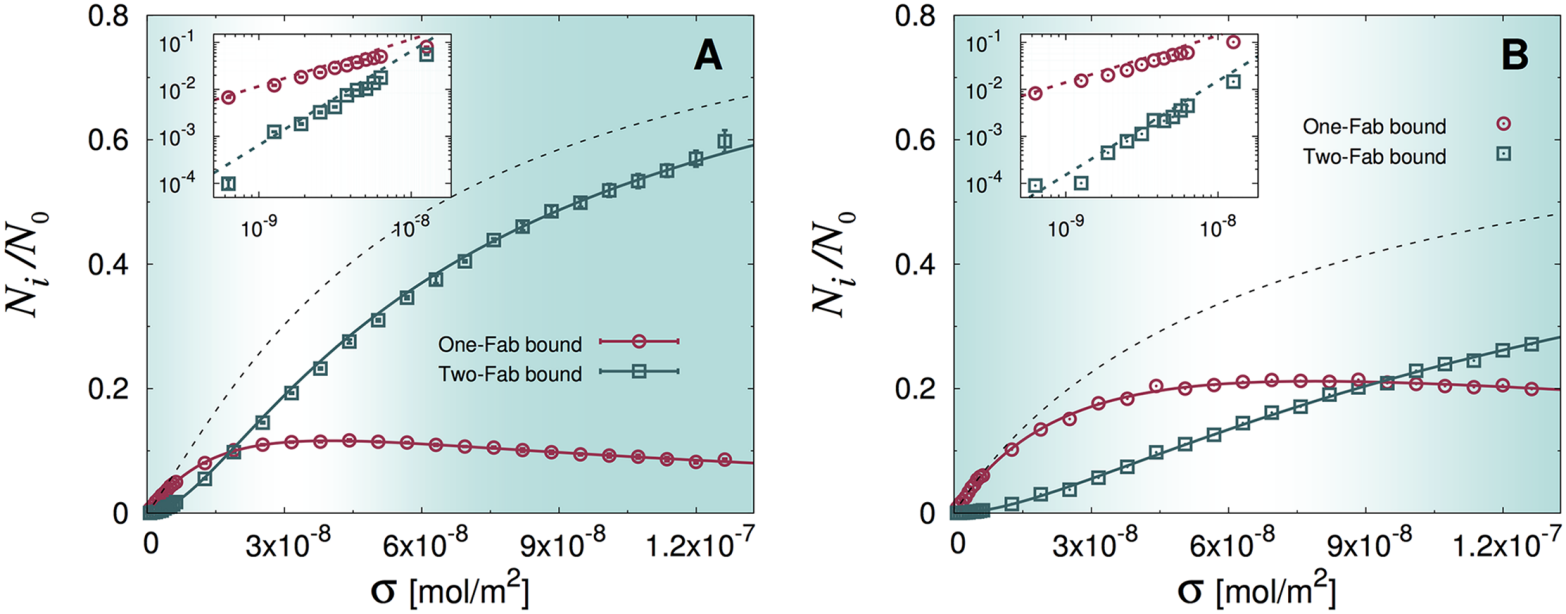
Plot of the stationary fractions of single-Fab and double-Fab bound antibodies versus antigen concentration (symbols) for the flexible (A) and rigid (B) IgG model and fits with the theoretical prediction, Eq (8) (solid lines). In both panels the black dotted lines show the total fraction of bound antibodies, (*N*_1_ + *N*_2_)/*N*_0_ and error bars are the corresponding statistical errors on the mean. The numerical results referring to the flexible IgGs (A) have been averaged over three different series of runs performed with different values of the solvent viscosity, as gauged by the parameter Δ*t* (see Methods). The insets show a close-up of the low *σ* region, highlighting the theoretical predictions *N*_1_ ∝ *σ*, *N*_2_ ∝ *σ*^2^ (see Eq (11)). Best-fit values of the floating parameters are reported in Table 1.

Binding of antibodies to surface-adsorbed antigens can be pictured as a two-step process, as sketched in Fig 3. First, antibodies diffusing in the bulk can encounter an epitope on the surface and bind to it through one of their Fab domains. As long as they remain bound, the second Fab domain has an opportunity to bind to any other epitope that lies within reach. As a consequence, the equilibrium surface concentration of bound IgG molecules, which increases with the surface concentration of antigens, is the sum of two contributions. This is illustrated in Fig 4(a). Antibodies bound through a single Fab dominate at low surface concentrations because there are few reachable epitopes for the second Fab domain. As *σ* increases, the number of double-Fab bound antibodies increases. At surface concentrations greater than ≈ 2 × 10^−8^ mol/m^2^, bivalent binding dominates because there are ample opportunities for the second Fab to bind through the fast exploration of a reduced volume. Concomitantly, the number of monovalent bound antibodies decreases as *σ* increases.

Within our scheme, the role of intramolecular flexibility can be easily addressed. We found that the ability of the three domains to change their relative angles strongly enhances their ability to bind antigens. *Rigid* antibodies with Fab-Fab and Fab-Fc angles restrained at 120° (see also Fig 2) exhibit a greatly reduced ability to bind antigens (Fig 4(b)). In particular, the population of bivalently bound IgGs is significantly depressed with respect to flexible antibodies, with the majority of antibodies being bound by a single Fab arm. Even at large surface concentrations *σ*, the equilibrium populations of single and double-bound rigid IgGs remain of comparable magnitude.

We conclude that double binding, which relies on the ability of single-bound antibodies to scan the surface in the proximity of the bound antigen, is greatly favored by the flexibility of the hinge connecting the Fab and Fc domains. This is an important conclusion, as it holds for any sort of multi-valent surface, such as the surface of large viruses [37]. In the following we describe a theoretical model that captures all the salient features of the IgG binding kinetics and reproduces perfectly our simulations. This model is a working tool that can be adapted to make quantitative predictions in many situations involving multivalent binding surfaces.

### Theoretical model

It is possible to shed further light on the observed binding equilibrium and to obtain more quantitative information by means of a simple analytical model. As illustrated in Fig 3, the surface concentration of single-Fab bound antibodies, *σ*_1_(*t*), evolves in time through exchange with antibodies in the bulk (volume concentration *ρ*_*B*_(*t*)), increasing upon IgGs binding at rate $k_{1}^{\text{on}}$ and decreasing at rate $k_{1}^{\text{off}}$ due to IgGs detaching from the surface back to the bulk. As well, *σ*_1_(*t*) varies in time through exchange with bivalent bound antibodies (surface concentration *σ*_2_(*t*)), decreasing because of the binding of the second Fab arm at rate $k_{2}^{\text{on}}$ and increasing because of its unbinding, at rate $k_{2}^{\text{off}}$. The corresponding rate equations read

$$\{\begin{array}{lll} \frac{\text{d}\sigma_{1}}{\text{d}t} & =2k_{1}^{\text{on}}\rho_{B}\sigma_{\text{av}}-k_{1}^{\text{off}}\sigma_{1}+2k_{2}^{\text{off}}\sigma_{2}-k_{2}^{\text{on}}\sigma \sigma_{1} & \left(1\right)\\ \frac{\text{d}\sigma_{2}}{\text{d}t} & =k_{2}^{\text{on}}\sigma \sigma_{1}-2k_{2}^{\text{off}}\sigma_{2}\; & \left(2\right)\\ \frac{\text{d}\rho_{B}}{\text{d}t} & =k_{1}^{\text{off}}\sigma_{1}-2k_{1}^{\text{on}}\rho_{B}\sigma_{\text{av}} & \left(3\right) \end{array}$$

where *σ*_av_(*t*) is the surface concentration of available antigens

$$\sigma_{\text{av}}\left(t\right)=\sigma -\left(\sigma_{1}+2\sigma_{2}\right)-\sigma \pi \ell^{2}\left(\sigma_{1}+\gamma \sigma_{2}\right)$$ (4)

The second term in the *r.h.s*. of Eq (4) takes into account the antigens that are *bound* to monovalently and bivalently attached IgGs. The third term accounts for antigens that are unbound but also unavailable, for *screened* by (hidden below) other antibodies fastened to neighbouring antigens. In order to describe such screening, we assume that a single-Fab bound IgG screens a circular patch of radius *ℓ* and that a double-Fab bound IgG screens a disk of radius $\ell \sqrt{\gamma }$. For what concerns the second binding, we assume that, once the first Fab is attached, the second Fab always sees the face-value surface concentration of antigens. This is due to the effective steric repulsion acting among IgGs bound on the surface, which makes the fraction of bound epitopes in an area within reach of the second Fab negligible.

The rate equations (1), (2) and (3) are obviously not linearly independent because the total number of antibodies in the simulation box is constant, *i.e*. *L*^3^ *ρ*_*B*_(*t*)+*L*^2^[*σ*_1_(*t*)+*σ*_2_(*t*)] = *N*_0_. The stationary rate equations have to be solved with the above constraint on the total number of particles. Setting *σ*_0_ = *N*_0_/*L*^2^, we obtain

$$\{\begin{array}{ll} (\sigma_{\text{0}}-\sigma_{\text{1}}-\sigma_{\text{2}})\left[1-\frac{\sigma_{\text{1}}+2\sigma_{\text{2}}}{\sigma }-\pi \ell^{2}\left(\sigma_{\text{1}}+\gamma \sigma_{\text{2}}\right)\right]=\left(\frac{K_{\text{1}}L}{2\sigma }\right)\sigma_{\text{1}} & \left(5\right)\\ \sigma_{\text{2}}=\left(\frac{\sigma }{2K_{\text{2}}}\right)\;\sigma_{\text{1}} & \left(6\right) \end{array}$$

where we have introduced the two dissociation constants

$$K_{i}\overset{\text{def}}{=}\frac{k_{i}^{\text{off}}}{k_{i}^{\text{on}}}\;i=1,2$$ (7)

**Fig 5:**
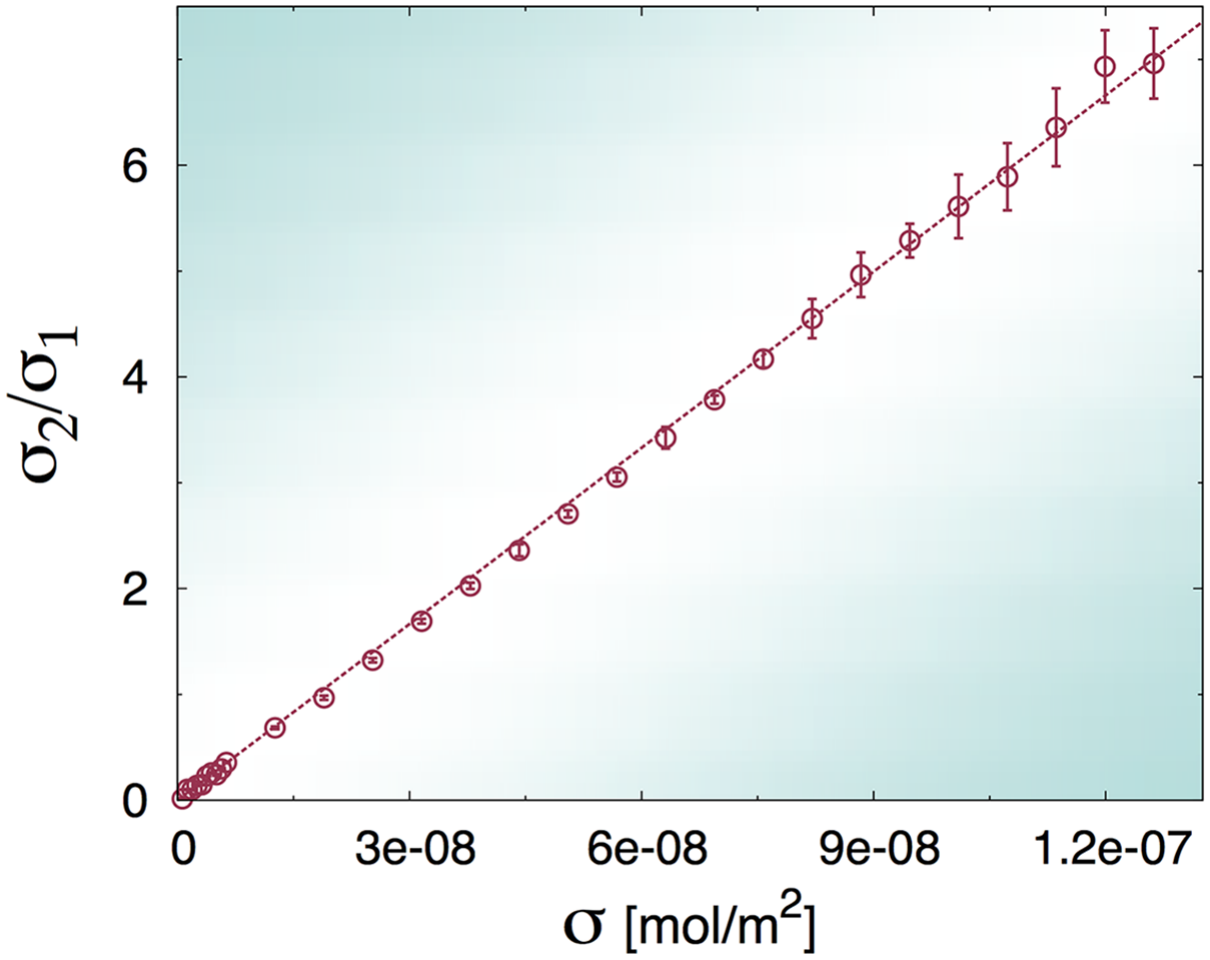
Plot of the ratio between the stationary concentrations of double-Fab to single-Fab bound antibodies computed from numerical simulations (symbols) and linear fit (dashed line). The numerical results have been averaged over three different series of runs performed with different values of the solvent viscosity (error bars are the corresponding statistical errors on the mean).

As a consequence of our assumption about the role of steric repulsion on the second binding, we see that our model predicts that the ratio *σ*_2_/*σ*_1_ should be linear with the antigen surface concentration *σ* with slope $1/2K_{2}$ (see eq. (6)). In Fig 5 we plot the results of EDBD simulations, which show excellent agreement with our model and thus confirm the soundness of our hypothesis.

A straightforward calculation shows that

$$\left\{\begin{array}{c} \frac{\sigma_{1}}{\sigma_{0}}=\left(\frac{2K_{2}}{2K_{2}+\sigma }\right)G\left(\sigma \right)\\ \frac{\sigma_{2}}{\sigma_{0}}=\left(\frac{\sigma }{2K_{2}+\sigma }\right)G\left(\sigma \right) \end{array}\right.$$ (8)

where

$$G\left(\sigma \right)=\frac{P\left(\sigma \right)+Q\left(\sigma \right)-\sqrt{{\left[P(\sigma )+Q(\sigma )\right]}^{2}-4Q\left(\sigma \right)}}{2Q(\sigma )}$$ (9)

with

$$\left\{\begin{array}{cc} & P\left(\sigma \right)=1+\frac{K_{1}K_{2}L}{\sigma (\sigma +2K_{2})}\\ & Q\left(\sigma \right)=\frac{2\sigma_{0}\left(K_{2}+\sigma \right)+\sigma_{0}\pi \ell^{2}\left(2K_{2}+\gamma \sigma \right)\sigma }{\sigma (2K_{2}+\sigma )} \end{array}\right.$$ (10)

Fig 4 shows that the model embodied by Eq (8) provides an excellent interpolation of the EDBD simulations. Best-fit values of the floating parameters are reported in Table 1 (see Methods for details on the fitting protocol). In our model, Fab arms are represented by prolate ellipsoids of aspect ratio 0.5, whose minor semi-axis is our unit of length. Therefore, in order to express our parameters in physical units, we have to estimate the length of a Fab arm, which is reasonably located between 6 and 7 nm. We note that the free energy changes associated with monovalent Fab (Δ*G*_1_) and bivalent Fab (Δ*G*_2_) binding equilibria estimated from our model compare rather well with the experimental values reported in [32], namely Δ*G*_1_ = −26.0 ± 0.4 kJ/mol and Δ*G*_1_ = −44.4 ± 0.7 kJ/mol. Our model predicts a decrease in free energy of the second binding event about twice greater than the first one (see last column in Table 1), in good agreement with the experiments (Δ*G*_1_/Δ*G*_2_ = 0.58). It is then apparent that our model correctly captures the physics of the double-step kinetics uncovered in the experiments reported in [32]. Incidentally, we note that the potential well describing the binding of a Fab tip with a surface-adsorbed hapten in our model could be easily tuned so as to obtain *exactly* the observed free energy changes as those measured in [32].

**Table 1:** Best-fit values of the floating parameters in the theories for the flexible (flexible) and rigid (rigid) IgG molecules with excluded-volume interactions and for the flexible IgGs that are transparent to each other on the surface (*ghost*). The theoretical models are Eq (8) (*flexible* and *rigid*) and the solution to Eq (14) (*ghost*). Indicated are also the free energy changes for the individual ligand-receptor binding steps obtained invoking ΔGi=kBT logKi at *T* = 300 K and assuming unit activity coefficients as in [32].

	*L*_Fab_ [nm]	$K_{1}\;[\text{M}]$	$K_{2}\;[\text{Mol}/{\text{M}}^{2}]$	*ℓ* [nm]	*γ*	Δ*G*_1_ [kJ/mol]	Δ*G*_2_[*kJ*/*mol*]	Δ*G*_1_/Δ*G*_2_
*flexible*	6	3.31×10^−4^	1.06×10^−8^	4.4	1.6	-19.9	-45.5	0.44
	7	2.09×10^−4^	7.77×10^−9^	5.1	1.6	-21.0	-46.3	0.45
*rigid*	6	2.74×10^−4^	5.45×10^−8^	6.2	1.0	-20.3	-41.5	0.49
	7	1.71×10^−4^	4.01×10^−8^	7.2	1.0	-21.5	-42.2	0.51
*ghost*	6	3.65×10^−4^	1.31×10^−8^	-	-	-19.6	-45.0	0.43
	7	3.59×10^−4^	8.79×10^−9^	-	-	-19.7	-46.0	0.43

As already observed, and in agreement with physical intuition, for rigid IgGs the second step in the association kinetics (binding of the second Fab) becomes strongly inhibited. This is now confirmed quantitatively by fitting the analytical model onto the simulations, which yields a five-fold increase of the dissociation constant $K_{2}$ (see Table 1). Furthermore, the fit highlights that the radius of the screening patch *ℓ* and *γ* depend as expected on the degree of flexibility of the antibodies. Monovalently bound rigid IgG molecules conceal a larger surface than flexible ones, *ℓ* being just about the length of one Fab arm. However, when the molecules establish a second binding on the surface, flexible IgGs screen a greater portion of the surface to other IgGs in the bulk than flexible single-Fab bound molecules do (about 1.6 greater). Rigid IgGs are seen to screen just about the same fraction of the active surface irrespective of their binding configuration.

We observe that for vanishing antigen concentration, our model predicts *N*_*k*_ ∝ *σ*^*k*^, *k* = 1, 2 (see insets in Fig 4). More precisely, a Taylor expansion for small values of *σ* shows that, for *σ* → 0

$$\left\{\begin{array}{cc} & \sigma_{1}≃\left(\frac{2\rho_{B}}{K_{1}}\right)\sigma \\ & \sigma_{2}≃\left(\frac{\rho_{B}}{K_{1}}\right)\frac{\sigma^{2}}{K_{2}}. \end{array}\right.$$ (11)

The above expressions can be used to compute a simple approximation for the fraction of bivalent bound IgG molecules, $f_{2}^{m}$,

$$f_{2}^{m}\equiv \frac{\sigma_{2}}{\sigma_{1}+\sigma_{2}}=\frac{\sigma }{2K_{2}+\sigma }$$ (12)

The fraction of bivalently bound *sites* on the surface, as measured *e.g*. in Ref. [32], $f_{2}^{s}=2f_{2}^{m}/\left(1+f_{2}^{m}\right)$, can be computed in a similar fashion, yielding

$$f_{2}^{s}\equiv \frac{2\sigma_{2}}{\sigma_{1}+2\sigma_{2}}=\frac{\sigma }{K_{2}+\sigma }$$ (13)

**Fig 6:**
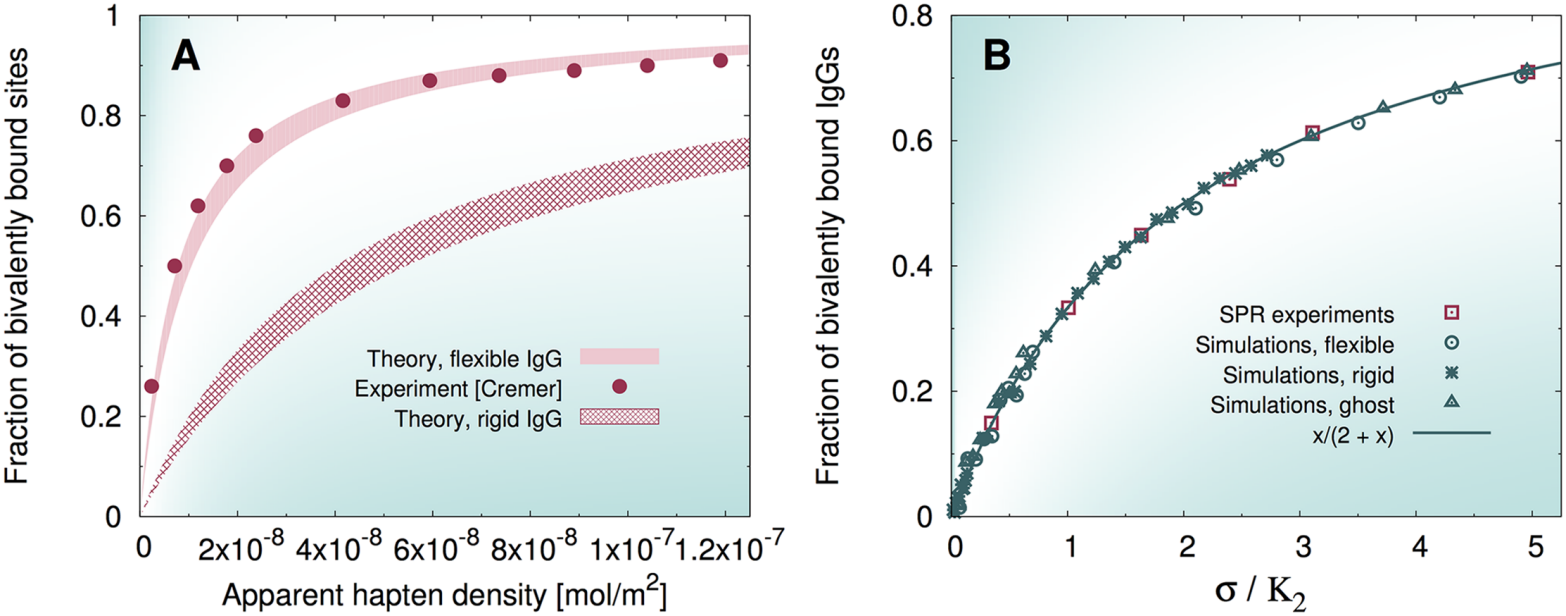
A) Plot of the fraction of bivalently bound sites $f_{2}^{s}$ as a function of the apparent (face-value) hapten density. Shaded regions correspond to the prediction of our model, Eq (8), for values of the Fab arm length between 6 and 7 nm (lower and upper curves delimiting the filled regions). Symbols are the experimental data reported in [32]. B) Plot of the fraction of bivalently bound *molecules* $f_{2}^{m}$ as a function of the rescaled surface density of haptens, $\sigma /K_{2}$. For the numerical data, the values reported in Table 1 were used. For the experimental data from [32], we used the value reported in the paper $K_{2}=0.69\times 10^{-8}$ mol/m^2^. The theoretical prediction (*x*/(2 + *x*)) is given by the Langmuir isotherm Eq (12).

Remarkably, a comparison of our results with the experimental data reported in the paper by Yang *et al*. [32], provides a further validation of our theoretical and numerical schemes. Fig 6(a) shows that, in order to capture the experimentally measured fraction of bivalently bound sites (or, equivalently, molecules), flexibility of IgGs is the primary requirement.

Interestingly, Fig 6(b) shows that when the surface density of haptens is normalized to the dissociation constant $K_{2}$, all different models and the experiments collapse on a single curve. This seems to suggest that $K_{2}$ contains all the relevant physics underlying the dynamics of the second binding. However, a moment’s thought is enough to realize that this conclusion is wrong. On the one hand, we have already seen that the separate binding profiles of single-arm and double-arm bound antibodies are profoundly influenced by the inherent flexibility of the molecules at high values of *σ* (see again Fig 4). On the other hand, rather surprisingly, it is evident from Fig 6(b) that the master curve is extremely well approximated by the low-*σ* prediction, Eq (12), over the whole concentration range. This indicates a compensation which is inherent to the specific normalization of the measures Eqs (12) and (13), that, being the latter *relative* indicators, conceal in the normalization the geometrical constraints that govern the large-*σ* regime. The important and non-obvious conclusion is that observables such as $f_{2}^{m}$ and $f_{2}^{s}$ cannot be used to disentangle the specific contributions of single and double binding.

Summarizing so far, we have introduced a kinetic scheme that describes both the kinetics of flexible and rigid IgGs. The crucial difference between the two models is that the dissociation constant for the second binding of rigid IgGs ($K_{2}=5.45\times 10^{-8}$ mol/m^2^) is about five times greater than the one for flexible IgGs ($K_{2}=1.06\times 10^{-8}$ mol/m^2^), which agrees well with the experimental value reported in [32], $K_{2}=0.69\times 10^{-8}$ mol/m^2^. Moreover, we have seen that looking at relative, normalized indicators, such as the fraction of bivalently bound antibodies, can be misleading, as relevant geometrical information is likely to be lost in the normalization.

### The role of excluded-volume interactions: simulations with *ghost* IgGs

The previous results clearly highlight the role of excluded volume on the antigen-antibody binding dynamics. To fully appreciate the impact of the mutual steric hindrance of antibodies on binding, we performed simulations with *ghost* flexible IgGs. In this scheme, the two Fabs and the Fc belonging to a given molecule interact with each other normally, so as to ensure the correct internal dynamics, but are transparent to domains belonging to other molecules. Therefore, a ghost IgG diffusing from the bulk will not see any of the available sites screened. Based on our theoretical arguments, one would expect that the binding equilibrium of *ghost* antibodies should be described by the solution of Eqs. (5) and (6) with *ℓ* = 0 (no steric obstruction). However, the rate equation for the second binding should also be modified due to the absence of steric repulsion on the surface. In fact, ghost IgGs trying to bind their second Fab are insensitive to the presence of neighboring bound IgGs. Thus, they are expected to probe the *available* density of binding sites and not the nominal one. In this case, the equilibrium surface densities should ensue from the following stationary equations (compare to Eqs. (5) and (6))

$$\left\{\begin{array}{cc} & \left(\sigma_{0}-\sigma_{1}-\sigma_{2}\right)\left(1-\frac{\sigma_{1}+2\sigma_{2}}{\sigma }\right)=\left(\frac{K_{1}L}{2\sigma }\right)\sigma_{1}\\ & \sigma_{2}=\left(\frac{\sigma }{2K_{2}}\right)\left(1-\frac{\sigma_{1}+2\sigma_{2}}{\sigma }\right)\sigma_{1} \end{array}\right.$$ (14)

**Fig 7:**
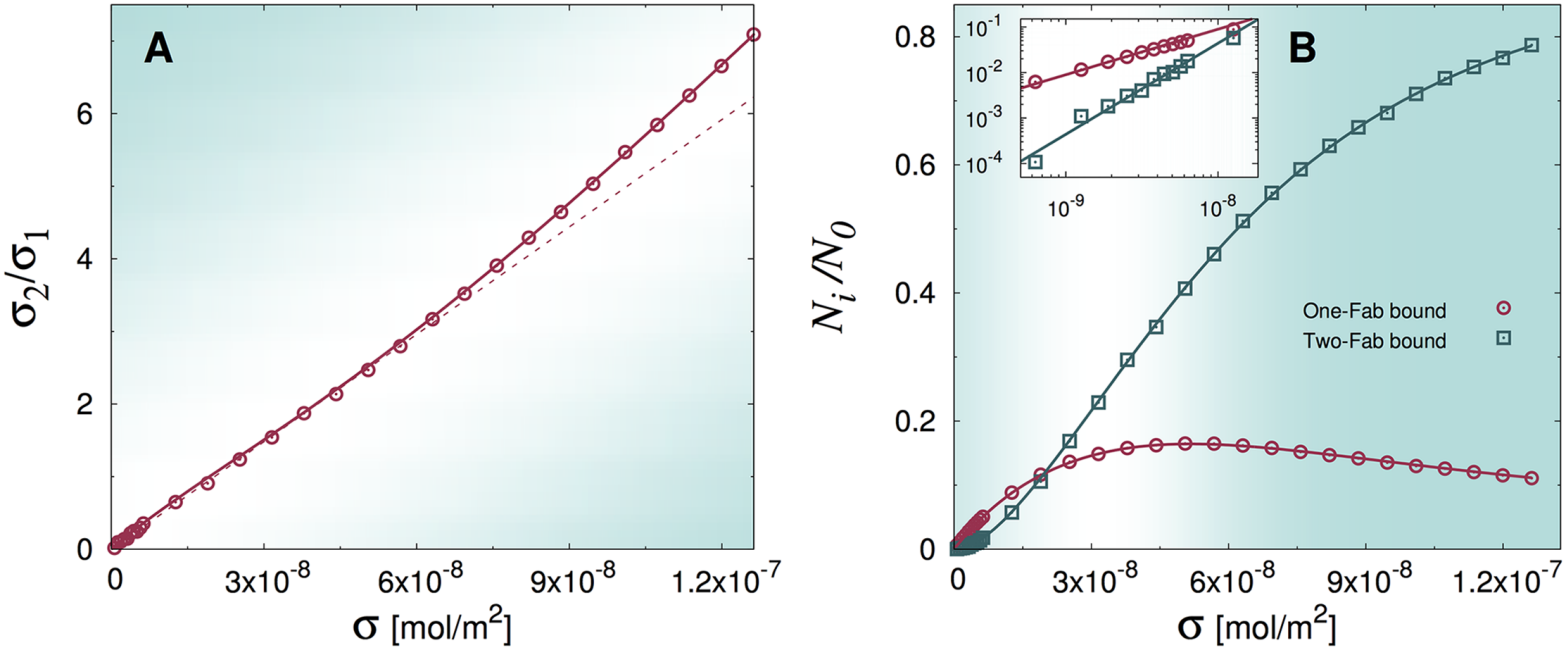
A) plot of the ratio between the stationary concentrations of double-Fab to single-Fab bound *ghost* antibodies computed from EDBD simulations (symbols). The dashed line is a linear fit performed in the interval 0 < *σ* < 5 × 10^−8^ mol/m^2^. B) plot of the stationary fractions of single-Fab and double-Fab bound antibodies versus antigen concentration (symbols) for the ghost IgG models. In both panels solid lines are fits to the solution of Eq (14). The inset shows a close-up of the low *σ* region, highlighting the fact that the theoretical predictions *N*_1_ ∝ *σ*, *N*_2_ ∝ *σ*^2^ (see Eq (11)) at low surface coverage hold unchanged and with similar equilibrium constants $K_{1}$ and $K_{2}$ irrespective of excluded-volume interactions. Best-fit values of the floating parameters are reported in Table 1.

The results of the simulations are reported in Fig 7. It is manifest that the solutions of Eq (14) provide a perfect fit to the simulations, confirming our physical intuition. At low hapten concentrations, there is virtually no difference between the binding of *ghost* and *hard-core* IgG molecules, because bound antibodies are, on average, far from each other. Hence *σ*_*k*_ ∝ *σ*^*k*^ at low values of *σ* (see also the inset in panel (b)). At larger hapten densities, instead, *ghost* molecules can bind significantly more than non-ghost ones (compare with Fig 4). This also appears as a marked deviation from the low-*σ* regime of linearity in the plot of *σ*_2_/*σ*_1_ vs *σ* (see panel (a)).

Interestingly, the equilibrium constants $K_{1}$ and $K_{2}$ do not change appreciably by eliminating excluded-volume interactions at the surface (see Table 1). As it is evident from the predictions Eq (11), the two dissociation constants are essentially fixed by the low-*σ* regime. Therefore, we draw the important conclusion that it is *flexibility* that gauges the magnitude of the dissociation constants (especially so concerning $K_{2}$). However, these do not tell the whole story, as the screening effect regulates the binding equilibrium at higher values of *σ*. This confirms that excluded-volume screening of otherwise free antigens on the surface is a contribution that is not negligible and that should mandatorily be taken into account in any modeling of the IgG binding dynamics on crowded surfaces.

## Discussion

In this paper we have introduced a coarse-grained model of immunoglobulin G (IgG) molecules, realized by fastening three ellipsoids with the proper aspect ratio together around a common hinge. The purpose of our study is to shed light on the role of the IgG flexibility and large size on its ability to bind to surface-absorbed antigens. Our coarse-grained (CG) model is conceived explicitly so as to reproduce the distributions of inter-domain angles measured by cryo-ET.

In our simulations, a large number of IgGs diffuse in a given volume and a binding equilibrium is reached with antigens adsorbed at a given density on the bottom surface. The equilibrium profiles of the surface concentrations of IgGs bound with one Fab and with both Fabs to the antigen-covered surface highlight the crucial role played by flexibility. When compared with antibodies frozen in an equilateral triangular configuration, fully flexible molecules demonstrate a much higher ability to bind with both Fabs. This is a direct result of a dynamic *search* process performed by the dangling second Fab of IgGs already bound with one arm. This capability of adapting to irregular antigen configurations is quenched in the rigid molecules, which are only able to bind bivalently when they happen to find two antigens lying at the appropriate distance matching their fixed Fab-Fab angular aperture.

In order to shed light on the observed binding equilibrium, we formulate a two-step kinetic model, where IgGs first bind from the bulk to the surface with one Fab (equilibrium dissociation constant $K_{1}$) and then double with the second Fab (equilibrium dissociation constant $K_{2}$). Importantly, our model not only includes the information on the *on* and *off* rates, but also accounts explicitly for an important geometrical constraint, namely surface *screening*. IgGs are large molecules and excluded-volume interactions, especially in the proximity of the antigen-covered surface, prove extremely important. This effect is two-fold. On the one side, (i) IgGs diffusing in the bulk *see* a number of available epitopes on the surface which is reduced with respect to the bare number of sites that are already occupied. In fact, a substantial number of non-bound antigens are nonetheless *de facto* unavailable as a result of the large size of IgGs bound in their proximity, which make them invisible to other antibodies in the bulk. Moreover, (ii) as a result of the IgG-IgG excluded-volume interactions at the surface, it turns out that the second Fab of a single-arm bound IgG always sees the face-value antigen concentration around, as it never gets to probe already occupied sites. These are too far away on average as a result of the effective repulsion among bound IgG molecules on the surface.

We conclude that the large and extremely flexible three-lobe conformation of IgGs is accurately designed to afford bivalent binding and at the same time take advantage of excluded-volume interactions to a maximum. This is probably the result of concurrent evolutive pressures towards smart antigen chasers capable of (i) bind *strongly*, *i.e*. with two binding sites (ii) bind *differentially*, *i.e*. bind to antigens of widely different sizes and (iii) bind *optimally*, *i.e*. maximize the number of bound molecules at a given concentration of target density.

Our model is in excellent agreement with surface plasmon resonance experiments of IgGs binding to surface-adsorbed haptens. We establish very clearly that flexibility is essential to reproduce the experiments (see Fig 6(a)). Furthermore, our theory allows us to isolate the second binding as the key factor that makes flexible IgGs much more powerful antigen binders. In fact, the dissociation constant measured from our simulations for rigid IgGs is five times larger than for the flexible ones.

A striking confirmation that the equilibrium constant for the second binding is the key factor can be gained by studying the equilibrium fraction of bivalently bound molecules (or, equivalently, sites). Once plotted against the antigen surface concentration rescaled by the dissociation constant $K_{2}$ (see Fig 6(b)), the three models, flexible, rigid and ghost fall on the same curve as the experimental data (where we have used the experimental dissociation constant). This remarkable fact proves very neatly that such *relative* measure conceals a great deal of the physics underlying the binding process.

The concealed information is instead conspicuous when one looks separately at the binding profiles, *i.e*. the average number of single-Fab (*N*_1_) and double-Fab bound (*N*_2_) IgGs against antigen concentration *σ*. More precisely, the low-concentration regime turns out to be the same in the three models, namely *N*_1_ ∝ *σ*, *N*_2_ ∝ *σ*^2^. At low antigen coverage, it makes no difference at all whether IgGs are able to stretch their second arm to get hold of neighboring haptens or whether they repel each other on the surface. Importantly, the low-*σ* regime fixes the two equilibrium constants $K_{1}$ and $K_{2}$, but obviously bears no sign of the screening effect. At increasing values of *σ*, the telltale signs of surface screening emerge clearly. Fully flexible molecules take advantage of the combined effects of their flexibility and mutual repulsion, which essentially makes haptens within reach of the second Fab always unoccupied on average. On the contrary, rigid IgGs remain largely unable to bind with both arms, despite their mutual exclusion, while ghost molecules take full advantage of their invisibility to bind with two Fabs, unphysically outperforming fully flexible antibodies at high densities.

We stress that our model includes in a natural way the kinetic parameters (*on* and *off* rates for the two binding events) and the key geometrical parameters. This is at variance with an existing model of IgGs binding to antigen-covered surfaces [33], which conceals the thermodynamic and geometrical information in one and the same parameter, namely an effective screening area. As such, our model provides a more accurate and valuable theoretical framework to interpret experimental profiles of antibodies binding to multi-valent surfaces in different contexts.

## Methods

### The CG Model

Each domain of the IgG is modeled as a rigid hard body. More specifically, the Fab fragments are modeled as two prolate ellipsoids while the Fc stem is modeled as an oblate ellipsoid. All the three fragments are hard ellipsoids of revolution, characterized by an aspect ratio *X*_0_ = *a*/*b*, where *a* is the length of the revolution axis and *b* is the length of the two other axes. For the Fab fragment we set *X*_0_ = 2, while for the Fc lobe we used the values *X*_0_ = 1/2, in agreement with the measured hydrodynamic radii [31]. All lengths are measured in units of the minor semi-axis of one IgG Fab, which is our non-dimensional unit (ndu). As in the real antibody the three domains are joined by a flexible hinge. This is realized by a spherical pivot, which is a particle with no steric hindrance (*i.e*. a *ghost particle*) and an attractive site of diameter 0.5 ndu, which forms an irreversible bond with the attractive sites placed on the three IgG fragments facing the hinge at a distance of 0.725 ndu from their surface (Fig 1). In general, in our EDBD simulations two attractive sites of diameter *δ*_1_ and *δ*_2_, which decorate two distinct particles, form a bond when their distance is less than (*δ*_1_ + *δ*_2_)/2.

The bond between the pivot and the sites located on the fragments is irreversible, *i.e*. it cannot be broken. The size of the pivot and of the attractive patches on the fragments have been chosen in such a way that an offset between the principal axes of the ellipsoids is accounted for, as deduced from the cryo-ET experiments of Ref. [28]. Each Fab’s tip is decorated with one *sticky patch*, whose center lies on its surface along the symmetry axis. Such patch may form a reversible bond with antigens placed on the bottom plane in the simulation box (cube of side *L* = 101.235 ndu). Antigens are modeled as attractive immobile sites. The diameter *δ*_*a*_ of the antigens is assumed to be 0.8 ndu and the diameter *δ*_*ab*_ of the patches on the Fab fragments is set to 0.6 ndu. The energy associated with the formation of a antibody-antigen bond is set to 10 *k*_*B*_*T*.

We performed event-driven Brownian dynamics (EDBD) simulations (see below) with three different variants of the IgG model illustrated above, which we refer to as *fully flexible*. In one variant, the ellipsoidal fragments belonging to different antibodies have no steric repulsion and can overlap. This model is referred to as *ghost*. In the second variant, the three fragments are fixed in a planar configuration where the three angles formed by the symmetry axes of the Fab and an axis perpendicular to the symmetry axis of the Fc fragment are all equal to 120°. This is achieved by three additional attractive sites located on the fragments, which form irreversible bonds. This model is referred to as the *rigid* model.

### Computer simulations

The IgG model we discussed above comprises excluded volume interactions, permanent bonds between attractive sites with infinite potential wells and reversible bonds implying finite-well potentials. A valuable tool to simulate such particles is offered by event-driven molecular dynamics (EDMD), especially in view of recent computational developments that allow one to simulate hard rigid objects (HRB) of generic shape decorated with attractive sites interacting with stepwise potentials [38–40]. In [41] an algorithm to perform Brownian dynamics of hard spheres is discussed. This algorithm has also been extended to anisotropic particles in [42]. Here we use this algorithm to perform event-driven brownian dynamics (EDBD) of the ellipsoidal particles (*i.e*. the Fab and Fc fragments) which form the IgG. The infinite-well sticky patches on the surface of an IgG keep it together, while the patches with finite-well potentials at the Fab tips bind to the surface-absorbed antigens. In our EDBD simulations we set a scaling time for the translational and angular velocities of the ellipsoidal fragments (see [42] for more details), which ensures that the typical displacement of their reversible sticky sites *δs* (i.e. those that give rise to antibody-antigen bonds) is smaller than the interaction range, *i.e*. *δs* ≪ (*δ*_*ab*_ + *δ*_*a*_)/2. The mass *m* of the three ellipsoids is equal and their moments of inertia are assumed diagonal and equal for all fragments. The latter choice is justified by the fact that the equilibrium properties of the antigen-antibody system do not depend on the dynamics used to evolve the IgGs in time.

### The kinetic model

We consider *N*_0_ antibody molecules in a volume *V* = *L*^3^ and *N*_*a*_ antigens (represented as spherical caps of radius *R*_*a*_) placed at random on the bottom face of the box, with a surface density *σ* = *N*_*a*_/*L*^2^. We denote with *n*_*B*_(*t*) the average number of antibodies in the bulk at time *t* and with *n*_*i*_(*t*) (*i* = 1, 2) the average numbers of antigen-bound antibodies featuring one-Fab bonds (*i* = 1) and two-Fab bonds (*i* = 2). We denote with capital letters, *N*_*B*_ = lim_*t* → ∞_ *n*_*B*_(*t*) and *N*_*i*_ = lim_*t* → ∞_ *n*_*i*_(*t*), the corresponding equilibrium, stationary, values. The corresponding surface densities are *σ*_*i*_(*t*) = *n*_*i*_(*t*)/*L*^2^ and bulk density *ρ*_*B*_(*t*) = *n*_*B*_(*t*)/*L*^3^. We model the binding kinetics as a two-step process as illustrated in Fig 3. An antibody in the bulk can bind with one Fab arm to a surface-adsorbed antigen with a rate $k_{1}^{\text{on}}$ and unbind with a rate $k_{1}^{\text{off}}$. An antibody that is already bound with a Fab can bind to an adjacent antigen with the second Fab with a rate $k_{2}^{\text{on}}$ and detach from the latter with a rate $k_{2}^{\text{off}}$.

### Fitting procedure

In order to fit the model parameters to the simulations for the rigid and flexible models with excluded volume interactions, we have first estimated $K_{2}$ by fitting the ratio *σ*_2_/*σ*_1_ versus *σ* (see eq. (6)). The parameters *γ* and *ℓ* turn out to be strongly anticorrelated. For this reason, we fitted *σ*_1_ vs *σ* by keeping *γ* fixed at regular values between 1 and 2 and let only *ℓ* and $K_{1}$ float. The dissociation constant $K_{2}$ was fixed at the value obtained from the previous fit. By doing this, we could select the best match (*γ*, *ℓ*) yielding the lowest value of the reduced chi square.

In the case of ghost IgGs the binding process to surface antigens is instead modeled by Eq (14) and the fitting procedure, which we adopted, was different. First, we solved analytically the two equations, thus obtaining *σ*_1_ and *σ*_2_ in terms of the fitting parameters $K_{1}$ and $K_{2}$. Note that there are three possible solutions of these equations but only one is physically meaningful. After that, we performed a simultaneous fit of *σ*_1_ and *σ*_2_ to numerical data from which we had an estimate of the equilibrium constants $K_{1}$ and $K_{2}$.
